# Group singing for health and wellbeing in the Republic of Ireland: the first national map

**DOI:** 10.1177/17579139221081400

**Published:** 2022-03-11

**Authors:** E Helitzer, H Moss

**Affiliations:** Master of Arts in Music Therapy, Irish World Academy of Music and Dance, University of Limerick, Limerick, Ireland; Senior Lecturer in Music Therapy, Irish World Academy of Music and Dance, University of Limerick, Limerick, Ireland

**Keywords:** group singing, singing, choir, social prescribing, health, wellbeing

## Abstract

**Aims:**

(1) To catalogue and map all singing for health and wellbeing groups in the Republic of Ireland (ROI); (2) determine how they prioritise health outcomes; (3) understand what they consider success; and (4) identify gaps in provision.

**Methods:**

A novel mixed-methods survey was distributed electronically through SING Ireland (the Choir Association of Ireland), artsandhealth.ie, and to 2736 potential stakeholders with links to singing for health and wellbeing and singing on social prescription (SSP) from October 2020 to April 2021. Thematic analysis was used to analyse four open-ended survey questions.

**Results:**

A total of 185 singing for health and wellbeing groups were identified, with varied representation in each of the ROI’s 26 counties. 35 groups were noted to have links to SSP. Gaps in provision for clinical and individual populations and for SSP were identified. Six themes related to the success of group singing for health and wellbeing programmes were determined: fostering and funding social and community connections; the people and the approach; enjoyment and atmosphere; musical and personal growth, programmatic structure and musical content; and the impact of Covid.

**Conclusion:**

The first-ever national mapping of group singing for health and wellbeing in the ROI, and one of few internationally, this study may serve as a roadmap for gathering information about existing singing for health and wellbeing provision and identifying geographical and clinical gaps internationally. Recommendations are included for future research to address gaps in provision, explore the feasibility of integrating SSP more widely and for further public health investment.

## Introduction

The evidence supporting the health and wellbeing benefits of group singing continues to expand.^1,2^ Increased feelings of social connection, happiness and rejuvenation,^3^ and enhanced memory and coping skills^4^ have been documented for individuals with dementia and their carers. Older adults living in the community reported lower levels of anxiety and depression,^5^ posture improvement,^6^ and diminished feelings of social isolation.^7^ Participation in an intergenerational choir has led to increased self-confidence for both older and younger members^8^ and lower anxiety for children at risk of academic failure.^9^

Group singing has provided positive experiences for individuals with cancer,^10^ respiratory wellbeing for people with chronic obstructive pulmonary disease (COPD),^11^ and improvements in vocal quality of life for individuals with Parkinson’s disease.^12^ Psychological and emotional benefits have been noted for the general public,^13^ homeless and marginalised individuals,^14^ and members of all-female singing groups.^15,16^ Adults with a chronic mental health condition and/or an intellectual or physical disability have experienced social, emotional, and practical gains.^17^ The positive impacts of singing in a workplace choir for staff-only groups^18,19^ and groups comprised of both staff and service users^20^ have also been documented.

Similar emotional, social, and practical gains have been experienced by Arts on Prescription (AoP) participants,^21^ a branch of social prescribing (SP). SP is an alternative or addition to pharmacological intervention wherein primary care physicians and healthcare professionals refer individuals with a range of needs to community link workers. Also known as SP coordinators, these individuals are knowledgeable about voluntary and community opportunities and connect referees with local, non-clinical, health, and wellbeing supports.^22^ The first documented SP project in the Republic of Ireland (ROI) took place in county Donegal in 2013.^23^

SP endeavours to combat inequity of access to healthcare by addressing economic and social factors, ‘Social Determinants’ that are the most significant predictors of health.^24^ A global issue, the ROI faces this challenge. Described as having a ‘two-tiered’ healthcare system, individuals who are able to purchase private insurance enjoy easier access to healthcare.^25^

Singing on social prescription (SSP) could serve as a tool to combat this inequity of access. First, a better understanding of provision and gaps in provision of group singing for health and wellbeing in the ROI is required.^26^ The creation of the first publicly accessible, living map of group singing for health and wellbeing in the ROI is a critical part of this investigation and will help overcome two identified barriers to the successful implementation of SP: inconsistent record keeping and the loss of organisational knowledge through frequent staff turnover.^27^

## Methods

This research was conducted by a small university team. The aims of this research were to catalogue and map all singing for health and wellbeing groups in the ROI, determine how they prioritise health outcomes, understand what they consider success, and identify gaps in provision. A novel mixed-methods survey comprised of 35 open- and closed-ended questions and informed by the Singing Europe Online Questionnaire^28^ was distributed electronically from October 2020 to April 2021 (Appendix 1). Data were collected on rehearsal/meeting locations, links to SP, choir demographics, leadership training, programmatic aims and priorities, and adaptations made for COVID.

The survey was distributed through the electronic mailing lists of SING Ireland (the Choir Association of Ireland) and artsandhealth.ie. 2736 potential stakeholders with links to singing for health and wellbeing and SSP were contacted via publicly accessible email addresses and social media, including university music departments, independent music schools, SP coordinators, health promotion officers, arts councils, organisations supporting individuals with disabilities, private and public hospitals, music therapy and community musician groups, community music and arts centres, libraries, branches of Music Generation, and branches of the Alzheimer’s Society. The list of potential stakeholders was compiled by the authors.

### Participants

A total of 224 responses were collected. Groups based outside of the ROI (*n* = 4) and respondents completing the survey on behalf of themselves as a solo singer (*n* = 1) were excluded. Informed consent was obtained to publicly share singing for health and wellbeing groups as a local resource. Individual participant information was kept confidential. Following deduplication, 210 survey responses remained for analysis, comprising 185 singing for health and wellbeing groups.

81.4% of respondents (*n* = 171) completed the entire survey. A total of 39 questionnaires were partially completed (83% completed, *n* = 4; 64%, *n* = 22; 28%, *n* = 12; 3%, *n* = 1). In instances of partially completed surveys, data pertinent to the primary aims of the study, such as name of singing group and location of service, were included.

### Procedure

Ethical approval for this study was received from the University of Limerick’s AHSS Research Ethics Committee. The questionnaire was created in Qualtrics. Potential informants received an email invitation with a link to the survey. Social media was utilised (i.e. Twitter and Facebook). Snowball sampling was employed as it has proven effective in gathering more obscure data.^29^ Survey data were analysed with SPSS 26 and only descriptive statistics were used.

Four open-ended questions were included:

1. Other priorities outside the seven options provided in the survey (Appendix 1).
2. What are the key ingredients that make this singing group successful?
3. What is one sentence that really gets to the core of how this programme prioritises the health and wellbeing of its participants?
4. Is there anything else you would like to add?

Qualitative responses were analysed using Braun & Clarke’s^30^ six-step framework for thematic analysis. The first author completed steps 1 to 4 (familiarising yourself with your data; generating initial codes; searching for themes; reviewing themes and generating a thematic map). Candidate themes and initial codes were reviewed by the second author, who confirmed the data linked to the themes identified. The two authors completed steps 5 (defining and naming themes) and 6 (producing the report) together. Themes were selected based on relevance rather than frequency counts, as this type of quantitative presentation can be limited in portraying the meaning and value of qualitative findings.^31^

## Results

### County

**Figure 1:**
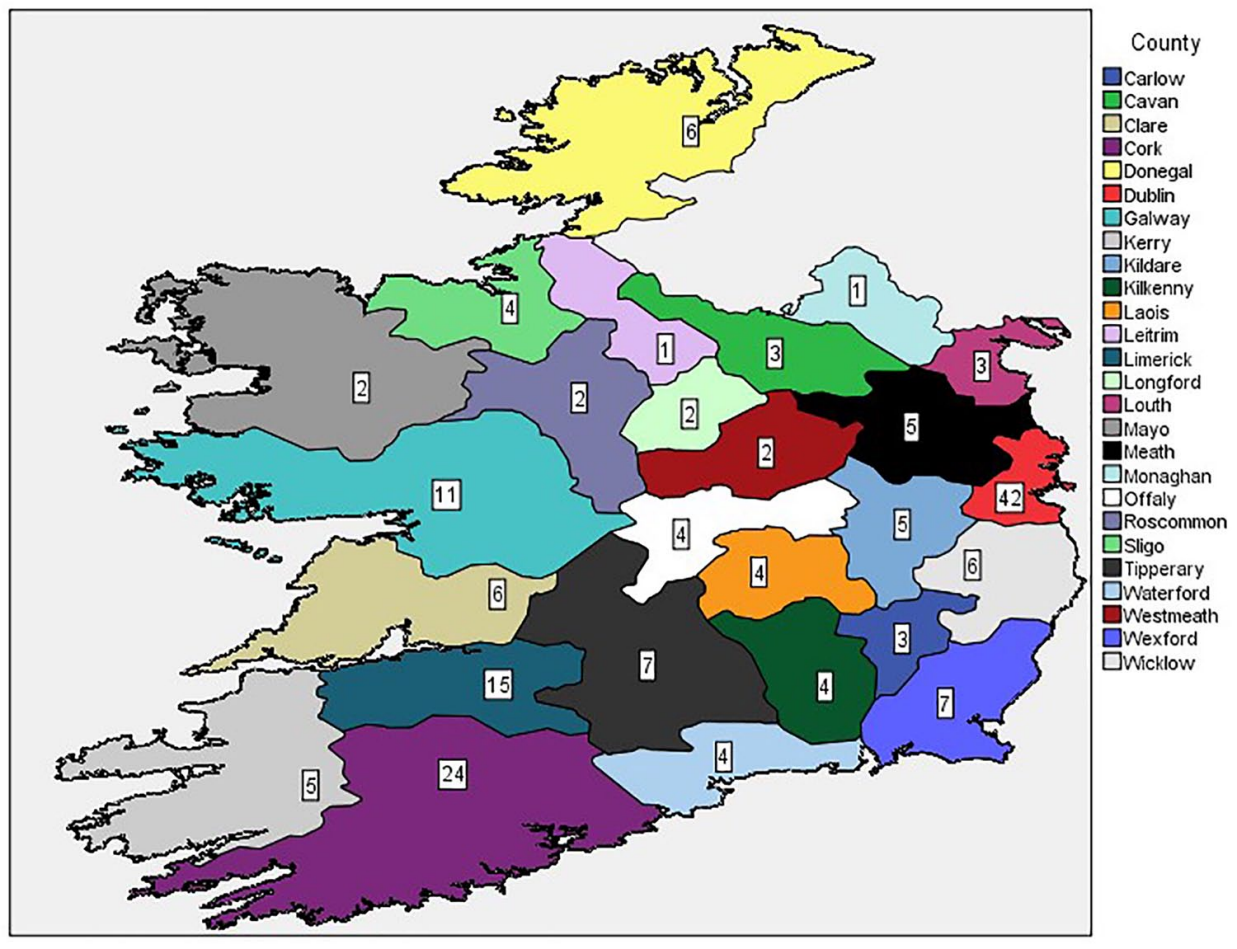
Map of singing groups per county.

**Figure 2:**
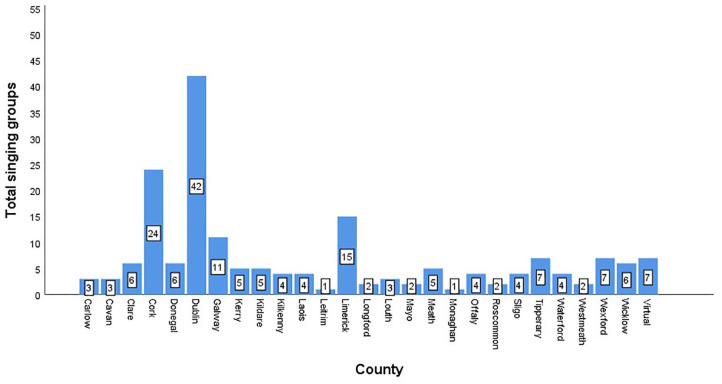
Bar graph of singing groups per county.

Singing for health and wellbeing groups were identified in each of the ROI’s 26 counties. In addition, seven nationwide, virtual groups were reported. County Dublin had the largest number of singing for health and wellbeing groups (*n* = 42). Counties Monaghan and Leitrim only had one each. A total of 92 groups (49.7%) were located in Dublin, Cork, Limerick, or Galway. Figures 1 and 2 outline total singing groups per county.

### Links to social prescribing

Respondents could note various sources of recruitment. In total, 33 groups across 13 counties, and 2 virtual groups, were considered to have links to SP as either a referral from a community link worker or healthcare professional was selected as one source of recruitment.

**Figure 3:**
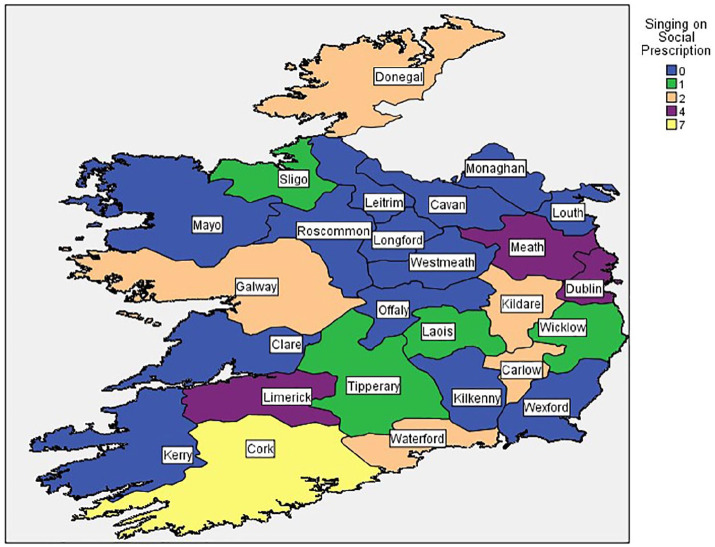
Map of singing groups with links to singing on social prescription by county.

Table 1 outlines total singing groups with links to SP per county. Figure 3 displays the same information with counties separated by colour. Counties in blue did not report any links to SP.

**Table 1:** Singing groups with links to singing on social prescription per county

County	Singing on SP groups per county
Cork	**7**
Dublin, Limerick, Meath	4
Carlow, Donegal, Galway, Kildare, Waterford Virtual	2
Laois, Sligo, Tipperary, Wicklow	1

### Choir demographics

Approximately 6265 individuals (4488 female, 1773 male, 4 do not identify as female or male) were members of singing for health and wellbeing groups. 61.4% (*n* = 3845) were members of groups open to anyone, including individuals with health conditions or additional needs, and 38.6% (*n* = 2420) were members of groups targeting a particular population. Demographics are detailed below.

#### Age and gender

A total of 158 groups reported singing group demographics for targeted (*n* = 80) and open groups (*n* = 79). The most common age range was 26–64 for both female (*n* = 2493) and male (*n* = 870) singers.

A breakdown of age and gender demographics is included in Table 2.

**Table 2:** Age and gender demographics of singing for health and wellbeing groups

	⩽15	16–25	26–64	65+	Total
Targeted groups (*n* = 80)					
Female	234	123	750	567	1674
Total groups	11	12	51	31	
Male	45	82	327	288	742
Total groups	8	12	46	32	
Do not identify as female or male	3	1	0	0	4
Total groups	1	1	0	0	
Open groups (*n* = 79)					
Female	126	90	1743	830	2814
Total groups	8	20	70	49	
Male	37	28	543	423	1031
Total groups	8	10	60	45	
Do not identify as female or male	0	0	0	0	0
Total groups	0	0	0	0	

#### Singing groups that target clinical or individual populations

A total of 171 groups specified being open to anyone (*n* = 81) or targeting a clinical, non-clinical, or individual population for membership (*n* = 90). Fifty-six of the 90 targeted groups, specifically for clinical or individual populations, are detailed below.

### Clinical and individual populations

#### Workplace choirs

The most common targeted singing groups were workplace choirs (*n* = 21) for current or retired staff and/or family members of hospitals, prisons, electronic companies and universities, and groups targeting older adults (*n* = 12), including: retirees (*n* = 4); older adults living in the community (*n* = 5); persons with dementia and their carers (*n* = 2); and older adults who utilise a day care centre (*n* = 1).

Eleven groups targeted individuals and supporters affected by illnesses or injuries such as cancer (*n* = 2); a mental health condition (*n* = 2); Parkinson’s disease (*n* = 2); stroke (*n* = 1); acquired brain injury (*n* = 1); COPD or other chronic lung diseases (*n* = 1). There were groups for individuals with physical disabilities (*n* = 2), adults with intellectual disabilities (*n* = 1), adults with intellectual disabilities and transition year students (*n* = 1), and adults with intellectual disabilities and/or a mental health condition (*n* = 1).

Less common were groups for individuals faced with the trauma of homelessness (*n* = 1); people in specific circumstances (e.g. recovering from mental health, addiction, facing difficulties) (*n* = 1); and residents of a particular area (*n* = 1).

**Figure 4:**
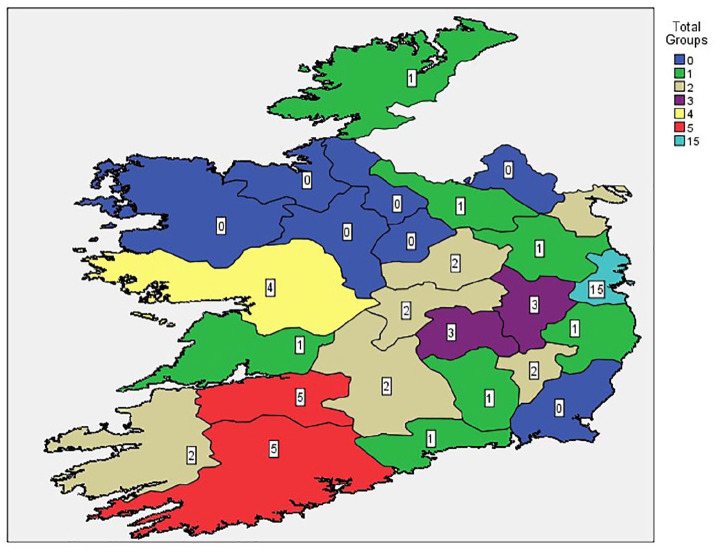
Map of singing groups for individual and clinical populations by county.

Provision of singing groups for individual and clinical populations is outlined in Table 3 and Figure 4. Eight counties indicated no provision and seven counties reported one singing group each.

**Table 3:** Provision of singing groups for individual and clinical populations per county

Provision per county	# of Counties	County name
0	8	Leitrim, Longford, Louth, Mayo, Monaghan, Roscommon, Sligo, Wexford
1	7	Cavan, Clare, Donegal, Kilkenny, Meath, Waterford, Wicklow
2	5	Carlow, Kerry, Offaly, Tipperary, Westmeath
3	2	Kildare, Laois
4	1	Galway, Virtual
5	2	Cork, Limerick
15	1	Dublin

#### Non-clinical populations

In total, 12 singing groups targeted young people or students and 11 were solely for men (*n* = 6) or women (*n* = 5). Appendix 2 includes a full list of clinical, non-clinical, and individual populations.

Auditions were not required for most singing groups (*n* = 137). A total of 138 (74.6%) groups met in a location easily accessible by public transportation.

#### Leadership training

In total, 75 choir leaders received specialised health and/or wellbeing training. 60% (*n* = 45) ran one of the 90 targeted groups; 40% (*n* = 30) ran one of the 81 open groups. A total of 64 types of relevant trainings were noted, most commonly: music therapy (*n* = 16); post-secondary degree in a music-related discipline (*n* = 8); community music (*n* = 5); and music teacher (*n* = 4). A full list of relevant trainings is included in Appendix 3.

#### Programmatic aims and priorities

Participants were asked to indicate if the following were programmatic priorities: a professional level of artistic production; singers’ general wellbeing; singers’ health; socialisation of singers; social integration; singing for pleasure/leisure; producing public concerts; and other.

A total of 131 groups responded. Singing for pleasure/leisure, socialisation, wellbeing, health, and social integration were top priorities. Producing public concerts and a high level of artistic production were slightly lower priorities. In total, 27 groups indicated ‘other priorities’, such as learning a skill or providing entertainment in nursing homes, as a primary aim.

#### Adaptations made for COVID

A total of 112 groups continued to meet after the Covid pandemic cancelled in-person singing groups. 87.5% (*n* = 98) incorporated a virtual component. Zoom was the most frequently used platform. Nine groups created virtual video projects, eight met outside when able, and one group leader made and sent CDs to those without Internet access.

#### Membership costs

In total, 68 (36.8%) groups were free to join. 50.8% (*n* = 94) had a variety of associated membership costs, ranging anywhere from €2 per rehearsal to €400/term. Most common were annual fees (*n* = 49), with an average cost of €175/year and a mode of €5/session (€260/year).

#### Rehearsal and performance

81.6% of choirs (*n* = 151) rehearsed weekly, most commonly for 1–2 h (*n* = 94) or 1 h (*n* = 52). A total of 156 individuals reported on performance schedule. Most performed between two and four times a year (*n* = 99) or annually (*n* = 20).

### Qualitative themes

In total, 386 responses were coded and analysed and 6 themes were identified: fostering and funding social and community connections; the people and the approach; enjoyment and atmosphere; musical and personal growth; programmatic structure and musical content; and the impact of Covid.

#### Fostering and funding social and community connections

Various types of local, national, and international connections were emphasised: reviving the singing tradition of a particular town, singing at religious services, and supporting individuals who may be isolated or vulnerable; performing tributes to Irish composers; linking in with the wider choral community.

Raising funds for local and national causes, and for singing groups, was also articulated. Support from local authorities, nongovernmental organisations (NGOs) and concert revenue, and donations from singers to pay group leaders were considered critical for success. Conversely, a lack of financial support was cited as a failing and disservice to the general population. Low financial expectations of singers encouraged membership and promoted inclusivity and accessibility:

*Regular visits to nursing/care homes locally promote interaction in a musical setting rather than performance … Weekly rehearsals are geared toward an annual presentation of about 16 choral … items and usually feature a tribute to an Irish composer …*

*Outlet for people who never meet with anyone[.] We look out for vulnerable and elderly people who have joined the [choir].*

*We all know music and song is good for your health. Choirs … need to be recognised for what they are, a very important health enhancing tool particularly in these strange times where the pandemic has impacted heavily on mental health across the country. These programmes should be main streamed with proper human resource and financial backing. This is not about professions, power, personal financial gain but rather this is about finding ways to positively improve the health of our people which has to be a priority particularly now.*

#### The people and the approach

The group leader, singers, and support staff were considered vital to the success of singing groups. Optimal group leaders were enthusiastic, professional, and committed, with excellent interpersonal and group facilitation skills. Professional training in music therapy, experience conducting choirs, and utilisation of the existing evidence base to support singers were instrumental.

Exemplary approaches were outlined: spreading a ‘we can do this’ attitude; motivating singers to do their best by combining hard work with fun; setting appropriate expectations; and supporting choral aims such as nurturing creativity, providing singers with a sense of purpose (e.g. concerts, original compositions), and promoting confidence and self-esteem.

Having enthusiastic, friendly, and supportive members with a shared interest (singing) contributed to group cohesion. Dedication to the group, both musically and as a peer support, was also noted. In some cases, though, performances were not a priority, and less emphasis was placed on rehearsing between sessions. In these instances, individuals weren’t ‘rehearsing’, simply meeting to gather and sing.

The importance of committee members, volunteers, and support staff was also highlighted, particularly in terms of organising finances, concerts, publicity, and providing technical support:

*… A competent and enthusiastic musical director is essential. A committee which manages finances, organization of concerts and publicity is also essential.*

*Living well with Parkinson’s Disease using the 5 Elements model developed by Move 4 Parkinson’s.*

*The social interaction and support between stroke survivors. It reduces feelings of isolation and self-consciousness as each member is a stroke survivor and will support and not judge his fellow member.*

#### Enjoyment and atmosphere

The singing environment was significant. Descriptors such as safe, relaxed, informal, joyful, friendly, non-threatening, supportive, and inclusive were used repeatedly. A home-like, community feel, supporting a sense of equality and belonging, regardless of age or ability, was depicted as ideal, particularly for reaching isolated or marginalised individuals, and those in need of an escape from stressful jobs or life situations. There were overlaps with the theme ‘fostering and funding social and community connections’ as environment could be key in enabling the creation of connections:

*It’s welcoming, everyone is treated equally and it’s an excellent way to escape from [life’s] problems.*

*To give people a safe, fun way of detaching from their worries and cares and promote positive mental health.*

*To offer a space for young mums to meet up, not enough support, and reach out to postpartum mums in our village community and for these mums social connections are key and time out of the week just for themselves without guilt.*

#### Musical and personal growth

Social, emotional, and musical opportunities and benefits were priorities and key ingredients of successful group singing programmes. Social benefits included the opportunity to make lifelong friends, be part of a choral family, and connect with others undergoing similar challenges. A safe, inclusive atmosphere allowed for emotional expression and exploration. Respondents noted the transformative impact singing together could have on members’ moods. Foci such as improved listening, communication, and social skills, the development of a routine and introduction to a, potentially, lifelong activity were outlined.

Group singing also provided musical opportunities: learning to use the voice as an instrument, receiving musical training and performing. A handful of respondents did note, however, that health and wellbeing were valuable by-products of group singing but not specific priorities. Collectively, the cultivation of transferable skills, in partnership with a supportive environment, helped build confidence and self-esteem:

*Creating a greater awareness of the voice as a musical instrument to provide a music education and life-long activity to the many children, young people and adults who never got or may never get the opportunity to play a music instrument due to personal circumstances, especially relating to availability of financial resources.*

*There is no doubt that many people arrive frazzled and leave cheerful.*

*Many older people were discouraged from singing in childhood. They find this a loss and need great encouragement. Also, as they are adults, they need a challenging musical experience so it is important to balance these two issues.*

#### Programmatic structure and musical content

Structure, organisation, and clear communication were considered necessary. Being mindful of singer’s availability and needs when choosing rehearsal time and location was crucial, as was repertoire selection. Singers were often encouraged to select repertoire, an intentional choice made by group leaders in order to empower and support members’ sense of ownership. Variety, genre, mood, and complexity of songs and the inclusion of warmups specific to singers’ health and wellbeing needs were also mentioned:

*[A]ccessible repertoire of meaningful songs and chants from all over the world …*

*The singing sessions combine vocal exercises and breath work to improve vocal problems with an additional focus on singing familiar and joyful songs to improve health and wellbeing.*

*Good boundaries and organisational skills. Creating a safe environment and having some housekeeping rules for everyone to understand.*

#### Impact of Covid

Group leaders were commended for their adaptability, creativity, and flexibility in keeping singers connected during Covid. Virtual efforts such as the creation of DVDs, remote music videos, and virtual singalongs were highlighted, with online sessions focusing on alternative ways of singing together. In some instances, the virtual aspect helped increase membership.

There were, however, drawbacks to connecting virtually, such as screen fatigue, decreased membership, preference for in-person meetings, and challenges to connect with individuals facing digital poverty. A longing to sing together again was noted, with the inability to meet creating a ‘vacuum in [our] lives’. Highly anticipated performances, gigs, and the potential for newer singing groups to grow were viewed as lost opportunities. These losses prompted a realisation of how significant group singing was for socialisation, peer support, relaxation, health and wellbeing, distraction, enjoyment, and providing a purpose. Concern was expressed over the future of group singing and whether it would ever feel safe again:

*Being adaptable especially now. Using creative ways to reach out to the Choir and to keep them all Singing. Our Remote Music Videos have been really successful and have also given the entire hospital community a positive distraction during this time.*

*The loss of the choir from covid is hard on all staff. At a time that staff need to [de-stress] and singing is not possible. We are working hard on risk assessment to try and restart the choir in 2021.*

*It has been an interesting development that our new term this September saw 4 new members join the choir, even though we can only rehearse online at present. This shows that people value being in a choir as they know it is good for their health, especially in this time of the global pandemic.*

## Discussion

This research contributes the first mapping of singing for health and wellbeing in the ROI and one of the first internationally. Several gaps in group singing for health and wellbeing provision were identified. Nearly half of all reported groups were located in just 4 of the ROI’s 26 counties. Interestingly, this provision is fairly consistent with the general population distribution. The percentage of singing groups per county mostly fell within 1% of that county’s share of the total population (Table 4) based on the 2016 census.^32^ Further research into aligning singing for health and wellbeing provision with clinical need is recommended, in addition to ensuring equity of geographical access for people with specific diagnoses. Partnerships with healthcare professionals, stakeholders and members of the Republic of Ireland’s publicly funded healthcare agency, theHSE, would be useful.

**Table 4:** Singing for health and wellbeing groups per capita

		% of 185	Share of total population 2016
Carlow	3	1.6	1.2
Cavan	3	1.6	1.6
Clare	6	3.2	2.5
Cork	24	13.0	11.4
Donegal	6	3.2	3.3
**Dublin**	**42**	**22.7**	**28.3**
Galway	11	5.9	5.5
Kerry	5	2.7	3.1
**Kildare**	**5**	**2.7**	**4.7**
Kilkenny	4	2.2	2.1
Laois	4	2.2	1.8
Leitrim	1	0.5	0.7
Limerick	15	8.1	4.1
Longford	2	1.1	0.9
**Louth**	**3**	**1.6**	**2.7**
**Mayo**	**2**	**1.1**	**2.7**
**Meath**	**5**	**2.7**	**4.1**
Monaghan	1	0.5	1.3
Offaly	4	2.2	1.6
Roscommon	2	1.1	1.4
Sligo	4	2.2	1.4
Tipperary	7	3.8	3.4
Virtual	7	3.8	N/A
Waterford	4	2.2	2.4
Westmeath	2	1.1	1.9
Wexford	7	3.8	3.1
Wicklow	6	3.2	3

Provision that is more than 1% below population share is shown in bold.

Gaps in provision for clinical populations were also identified. Eight counties did not report any provision for clinical or individual populations; an additional seven only had one singing for health and wellbeing group each. This gap is significant considering that in 2021 there were 170,000 individuals living with or beyond cancer in Ireland,^33^ yet only two singing groups for individuals with cancer were reported. Further research on addressing gaps in provision for clinical and individual populations would be beneficial, particularly through SSP initiatives.

18.9% (*n* = 35) of singing groups indicated possible links to SSP. Considering the potential cost-effectiveness of both group singing and SP,^34,35^ it would be valuable to explore the feasibility of expanding SSP in the ROI by identifying current models of best practice.

Overall, far more women participated in singing for health and wellbeing than men. Of the 6265 singers reported, 71.6% were female, highlighting a common gender imbalance within singing groups.^36^ Further research and consultation with experts are recommended to understand the needs of men, people from culturally diverse backgrounds and why particular populations are less prone to participate in such singing groups.

A wide range of professions and trainings deemed relevant for running a singing for health and wellbeing group confirms literature in the field.^3^ A lack of heterogeneity regarding training and overall programmatic structure is also notable. Both merit further research.

## Conclusion

While there is a growing body of evidence around the health and wellbeing benefits of group singing, there is a paucity of national mapping investigations. This study outlined the motivation, methodology, and findings of the first-ever national mapping of group singing for health and wellbeing in the ROI and one of few internationally. It may serve as a roadmap for gathering information about existing provision and identifying geographical and clinical gaps internationally.

The authors hope this study will serve as a springboard for the continued growth and development of a national and international map of group singing for health and wellbeing, a potentially instrumental resource for singers, referrers, and stakeholders.

Several gaps in provision have been identified for SSP and various populations. Recommendations are included for future research to address these gaps and explore the feasibility of integrating SSP more widely. The authors hope these findings will inform healthcare policy and encourage greater financial allocation for group singing for health and wellbeing. The evidence to date supports singing for health and wellbeing as a potentially cost-effective intervention.^34^ Further public health investment and research is warranted.

## Appendix 1

### Online survey

Mapping singing for health and wellbeing in the Republic of Ireland

**CONTACT**

Name of choir or singing group:______________________________________________________

Location of service: ________________________Year Established: ________________________

Contact Email address: ________________________ Contact Phone number: __________

Website:________________________________________________________________________

Is this service affiliated/part of another organisation? __Y __N

If yes, which organisation: ________________________________________________

And does this organisation run other singing groups? __Y __N

**ABOUT YOUR GROUP**

Please estimate the total number of choir members at the start of 2020 (pre-Covid).

**Table:**

	<15 years old	16–25	26–64	65+
Female				
Male				
Do not identify as female or male				

Is this singing group open to any member of the community or does it target a specific population?

__Open to anyone – come one come all!
__Targets a specific population

Do participants need to audition to join? __Y __N

If a specific population is targeted (e.g. individuals with dementia), how would you describe the target population? __________________________________

Ordinarily, the singing group meets/rehearses:

__Weekly
__Monthly
__Occasionally as time allows
__Only to prepare for specific performances
__Other, please describe: ________________________________________________

Rehearsals typically last:

__An hour
__1–2 h
__2 h+

Are rehearsals held in a location that is easily accessible by public transportation?

__Y __N

Since Covid, the singing group:

__No longer meets
__Meets in a different way (i.e. virtually, over the phone). Please describe: ________

Has the choir leader received any specialised training specifically linked to supporting the health and wellbeing of this target population? (e.g. training in music therapy, group facilitation, health and wellbeing) __Y__N__N/A

If yes, please describe: ________________________________________________

Is there a fee to join this group? __Y __N

If yes, what is the approximate annual cost? ________

**RECRUITMENT**

How have group members learned about this opportunity? (please select all that apply)

__Referral from healthcare professional (i.e. social prescription, GP, mental health professional)
__Referral from community link worker (i.e. social prescribing coordinator, community health worker, community volunteer)
__Independently sought out choir
__Word of mouth
__Other (please describe):________________________________________________

**PERFORMANCE**

Does the singing group perform to an audience?

__Weekly
__Monthly
__Between two and four times a year
__Once a year
__Never

**Choir aims**

**Please note with an ‘X’ whether you agree or disagree that the aims listed below are priorities for this particular group, and rank in order highest (1) to lowest (7) priority.**

**Table:**

Aim	Strongly disagree	Disagree	Neutral	Agree	Strongly Agree
A professional level of artistic production					
Singers’ general wellbeing					
Singers’ health					
Socialisation of singers (making friends and building relationships)					
Social integration (singers of different generations or cultural background)					
Singing for pleasure/leisure					
Producing public concerts					
Other (please explain below)*					

*Other priorities________________________________________________

What are the key ingredients that make this singing group successful?__________________________________________________________________

What is one sentence that really gets to the core of how this programme prioritises the health and wellbeing of its participants? ________________________________________________

Has the programme been evaluated in some capacity? __Y__N

Is there anything else you would like to add?________________________________

Name of individual completing survey:________________________________

Contact email:_________________________Contact phone number: ________________________________

Would you be willing to participate in a short conversation to follow-up on this survey? __Y __N

Would you like to receive a summary of the results of the study? __Y__N

LOCAL RESOURCES

We are aiming to compile a list of singing resources around the country. Are the details you listed previously the details you would like us to use?

__Y __N __If no, please enter details here: ________________________________

## Appendix 2

**Table:** List of non-clinical, clinical and individual populations

	*n*
Clinical and Individual Populations	
Workplace Choirs	
Hospital staff – current and retired	3
Hospital staff	2
Hospital staff – current, retired and family	1
HSE Staff	3
Dun Laoghaire Rathdown County Council current and retired staff	2
HSE Staff – current and retired	1
HSE and Tusla staff	1
Staff, service users and family/friends of people within the mental health sector of HSE, Mid West	1
Children’s Health Ireland Staff	1
Cork Prison Male Staff	1
Galway Mayo Institute of Technology Staff	1
Westmeath County Council Staff	1
Medtronics – current and retired staff	1
Volunteers supporting individuals with mental health condition	1
Volunteers in the community, community members	1
Older Adults	
Retirees	5
Aged 55 and older, community members with dementia and their carers	2
Aged 50 and older	1
Aged 55 and older	1
Older adults using a particular day centre and/or living in a residential unit	1
Senior citizens	1
Primarily older adults in the community	1
Individuals Affected by Illness or Injury	
Individuals affected by cancer	2
Individuals with Parkinson’s Disease and their carers/family/friends	2
Individuals with a mental health condition	2
People with COPD or other chronic lung disease	1
Individuals affected by stroke	1
Individuals with an acquired brain injury	1
Family carers	1
Adults with physical disabilities	2
Adults with intellectual disabilities and Transition Year Students	1
Intergenerational	2
General health and wellbeing for adults	1
Marginalised Community	1
people availing of a Galway Simon Project service/affected by the trauma of homelessness	1
New migrants to Ireland	1
Individuals in specific circumstances e.g. recovering from mental health, addiction, facing difficulties – as outlined by The Lantern Project	1
Residents of the Tullow Road area of Carlow Town	1
Non-Clinical	
Gender	
Men	5
Members of Ballina Men’s Shed	1
Women	4
Women from different nationalities	1
Young People/students	12
Adults 18+	5
Adults 16+	1
Students and alumni of Galway Mayo Institute of Technology	1
Professional singers	1
Irish speakers	1
Ukulele players who sing	1
Missing	1

## Appendix 3

**Table:** Relevant health and wellbeing professions and trainings

Profession	Music Therapist	13
	Community Musician	5
	Music Teacher	4
	Professional Musician	3
	Arts and Health Professional	2
	Works at a university	2
	Academic, Arts Organisation Professional, Arts Practitioner Researcher, Community Development, Community Musician/Music Therapist, Counselling, Medical Doctor, Mental Health Professional, Occupational Therapist, Psychotherapist, Rehabilitation Officers/Social care, background in music, Social Therapist, Speech and Drama Teacher/background in music, Speech and Language Therapist, Stroke Support Group Coordinators	1
Educational Background		
Obtained or Pursuing Diploma or Certificate	Certificate in Music Therapy	2
	Adult Training and Development, Community and group music teaching, Community Music, Homoeopathy, Certificate in Music Therapy	1
Bachelor’s Degree	Music	3
	Psychology, Training and Education, Unspecified	1
Obtained or pursuing postgraduate Degree	Arts, Chaplaincy, Choral Studies, MA in Vocal Pedagogy in Singing for health and wellbeing, Music, Music Education, Theology	1
Trainings	Experience in Choral Conducting	4
	Dementia Training	2
	Group Facilitation	2
	Health and wellbeing	2
	Music Network Training	2
	MusicAlive	2
	Musicians Without Borders training	2
	Natural Voice Network	2
	CALM–Music and Health musicians training, Choral Workshops, Estill voice training, Holistic Wellness Tutor, Kid’s Classics music in healthcare training, Kodály music education method, Meditation, Mindfullness, Purple House Cancer Support Training, Sing to Beat Parkinson’s, Singing for Lung Health, Singing for Parkinson’s, Singing for the Brain, Social & Health Education Project Trainings, Cork, Sound & Music for Healing and Personal Development, Trauma-informed music facilitation, Vocal Health Education, Vocal Health First Aid, Wellbeing	1
